# Risk factors of severe hospitalized respiratory syncytial virus infection in tertiary care center in Thailand

**DOI:** 10.1111/irv.12793

**Published:** 2020-08-12

**Authors:** Puneyavee Aikphaibul, Tuangtip Theerawit, Jiratchaya Sophonphan, Noppadol Wacharachaisurapol, Nattapong Jitrungruengnij, Thanyawee Puthanakit

**Affiliations:** ^1^ Department of Pediatrics Faculty of Medicine Chulalongkorn University Bangkok Thailand; ^2^ Center of Excellence for Pediatric Infectious Diseases and Vaccines Faculty of Medicine Chulalongkorn University Bangkok Thailand; ^3^ The HIV Netherlands Australia Thailand Research Collaboration (HIV-NAT) The Thai Red Cross AIDS Research Centre Bangkok Thailand; ^4^ Clinical Pharmacokinetics and Pharmacogenomics Research Unit Department of Pharmacology Faculty of Medicine Chulalongkorn University Bangkok Thailand

**Keywords:** bronchodilator, cirrhosis, community acquired, death, hospitalization, nosocomial infection, respiratory syncytial virus

## Abstract

**Aim:**

To determine factors associated with severe hospitalized Respiratory syncytial virus (RSV)‐associated LRTI and to describe management in tertiary care center.

**Methods:**

Retrospective medical record review was conducted among children under 5 years old hospitalized with RSV‐associated LRTI at King Chulalongkorn Memorial Hospital. Severe RSV‐associated LRTI was defined as death, mechanical ventilator, or positive pressure ventilation use, prolonged hospitalization >7 days. Factors associated with severe RSV were analyzed using univariate and multivariate logistic regression.

**Results:**

From January 2011 to December 2016, 427 children were hospitalized. Median age was 10 months (IQR 4.2‐23.0). One hundred seventy‐four (41%) patients had severe RSV (11 deaths, 56 mechanical ventilators, 19 positive pressure ventilation, and 88 prolonged hospitalization). Factors associated with severe RSV were chronic lung disease (aOR 15.16 [4.26‐53.91]), cirrhosis/biliary atresia (aOR 15.01 [3.21‐70.32]), congenital heart disease (aOR 5.11 [1.97‐13.23]), chemotherapy (aOR 4.7 [1.34‐16.56]), and pre‐term (aOR 2.03 [1.13‐3.67]). Oxygen therapy was mainly low flow oxygen delivery. 88% of cases received bronchodilator. Parenteral antibiotics were prescribed in 37.9% of cases.

**Conclusions:**

Children with co‐morbidities have higher risk of severe RSV‐associated LRTI. More than two‐third of patients received bronchodilator, of which was not recommended by American Academy of Pediatrics. The specific treatment and prevention for RSV are urgently needed.

**Abbreviations**
AAP: American Academy of Pediatrics
aOR: adjusted odd ratio
BIPAP: Bi‐level positive airway pressure
CI: 95% confidence interval
CLD: chronic lung disease
CPAP: continuous positive airway pressure
HIV: human immunodeficiency virus
ICD‐10: International Classification of Disease 10th revision
IQR: interquartile range
IRB: Institutional review board
KCMH: King Chulalongkorn Memorial Hospital
OR: odd ratio
RSV Ag: respiratory syncytial virus Antigen
RSV: respiratory syncytial virus

**Key notes**

- Factor associated with severe hospitalized RSV were pre‐term, infant, co‐morbidity of cardiopulmonary disease, on chemotherapy, and cirrhosis/biliary atresia.
- Low flow oxygen use was main oxygen therapy. Most of patients were receiving bronchodilator and antibiotics.

## 1 INTRODUCTION

Respiratory syncytial virus (RSV) infection is well‐known for childhood hospitalization from acute lower respiratory tract infection (LRTI) especially young children in developing country.^1^ About 200 000 deaths occurred from RSV‐associated LRTI worldwide each year.^2^ High incidence of hospitalization due to RSV‐associated LRTI is associated with higher risk of in‐hospital mortality.^3^ In Thailand, incidence of acute RSV‐associated LRTI in year 2008‐2011 was 85/100 000 persons/year particularly children age 5 years.^4^ Seasonality of RSV was usually occurred during July to October.^4^, ^5^, ^6^

After 3‐7 days of incubation period, patient with RSV‐associated LRTI may have common symptoms such as fever, runny nose, cough, wheezing, or difficult breathing.^2^ Confirmation usually be done by RSV Ag rapid test on sample collected from nasopharyngeal swab.^3^ Risk factors for severe RSV‐associated LRTI have been described in many studies such as infant, prematurity, cardiopulmonary disease, bacterial co‐infection, proximity of birth to RSV season, exposure to environmental smoking, male sex, and familial atopy.^7^, ^8^, ^9^, ^10^, ^11^ Also laboratory finding, thrombocytosis at time of diagnosis is one of predictors for severe RSV‐associated LRTI.^12^ Nosocomial infection is one of the factors associated with intensive care unit and mortality.^13^, ^14^ Currently, there is immunoprophylaxis for RSV, a humanized monoclonal antibody called palivizumab, and it has been used in many developed country but only for pre‐term infant gestational age <29 weeks, infant with chronic lung disease, and children with hemodynamically significant heart disease.^7^ However, the availability in developing countries is limited.

As RSV‐associated LRTI responsible for majority of acute lower respiratory tract infection, many treatment modalities were used. For RSV‐associated LRTI, American Academy of Pediatrics (AAP) recommended treatment is mainly supportive (oxygen supplement to maintain oxygen saturation and fluid therapy), and other treatments such as bronchodilators, epinephrine, corticosteroid, hypertonic saline, and antibiotics are not useful.^7^ However, there is limited information of pattern of treatment in developing country including Thailand.

This study aimed to determine factors associated with severe hospitalized RSV‐associated LRTI and to describe pattern of management in tertiary care center in Thailand.

## 2 MATERIAL AND METHODS

This retrospective descriptive study was conducted at King Chulalongkorn Memorial Hospital (KCMH), a tertiary care 1500‐bed university hospital in Bangkok, Thailand. Pediatric department of 200 beds usually had an average 6500 outpatient visits and 1000 inpatient hospitalized per month. Study population included patient age 0‐5 years old admitted in KCMH during January 2011‐December 2016 with diagnosis of RSV‐associated LRTI. Case assessment was performed by either ICD‐10 from hospital summary discharge and/or positive laboratory test for RSV identified from microbiological database either rapid antigen test using QuickNavi™ from Denka Seiken Co., Ltd or PCR test for RSV using Simplexa™ from DiaSorin Molecular LLC. Data were extracted from medical record to case record form after approved from Institutional Review Board (IRB) of Faculty of Medicine, Chulalongkorn University (IRB No. 243/60).

Patient who was diagnosed of RSV‐associated LRTI as the cause of hospitalization was recorded as community‐acquired RSV‐associated LRTI patients. Severe RSV hospitalization was defined as patient who needed intubation or non‐invasive positive pressure ventilation (such as high flow nasal cannula, continuous positive airway pressure [CPAP], bi‐level positive airway pressure [BIPAP]), or prolong length of hospital stay in community‐acquired RSV‐associated LRTI (more than 7 days) and in‐hospital death which would be discussed separately. Co‐morbidity or underlying disease was condition of patient prior to RSV‐associated LRTI. Chronic lung disease such as bronchopulmonary dysplasia was determined regardless of home oxygen use. Immunocompromised status included congenital immunodeficiency, HIV, on immunosuppressive medication, or chemotherapy. Perinatal history, pre‐term was defined as patient born at gestational age <37 weeks. Low birthweight was <2500 g. Physical examination was from initial presentation. Diagnosis of RSV was made by attending physician. Lower respiratory tract infection was bronchitis, bronchiolitis, and pneumonia. Thrombocytosis was defined as platelet count more than 400 000/µL. Oxygen therapy was analyzed from maximum oxygen delivery use as low flow oxygen, high flow oxygen, non‐invasive positive pressure ventilation, and invasive ventilation, respectively.

Factors associated with severe hospitalized community‐acquired RSV‐associated LRTI included host factors and clinical presentation. Host factors such as demographic data, perinatal history, and underlying disease were collected. Clinical presentation determined as presenting symptoms and initial physical examination, and initial laboratory findings determined as complete blood count. Outcome of patient at discharged was categorized as discharge without readmission or readmission within 1 month with the same diagnosis or in‐hospital death.

For hospital‐acquired RSV‐associated LRTI, patient who was diagnosed of RSV‐associated LRTI later during hospital stay, after 72 hours after admission, was recorded as hospital‐acquired RSV‐associated LRTI. Data were extracted and compared with community‐acquired RSV‐associated LRTI.

### 2.1 Statistical analysis

The primary objective of this study was to determine factors associated with severe hospitalized RSV‐associated LRTI such as underlying disease, perinatal history, clinical presentation, and laboratory findings. Secondary objective was to describe pattern of management in hospitalized RSV infection including oxygen therapy, intravenous hydration, bronchodilator nebulizer, and antibiotics use. Factors associated with severe hospitalized RSV‐associated LRTI were analyzed from community‐acquired group. For baseline characteristics of all patients, severe hospitalized RSV‐associated LRTI were compared with not severe RSV infection using chi‐square for categorical data and Wilcoxon rank‐sum test for continuous data.

Factors associated with severe RSV‐associated LRTI were analyzed using univariate and multivariate logistic regression if *P*‐value < .1. Management pattern for RSV infection in study was described as percentage and frequency for categorical data and median for continuous data. Data were managed by Statistical Package for the Social Sciences (SPSS) version 22.0 and analyzed be STATA version 15.1.

## 3 RESULTS

### 3.1 Study population: manifestation and outcome

#### 3.1.1 Demographic data

**FIGURE 1:**
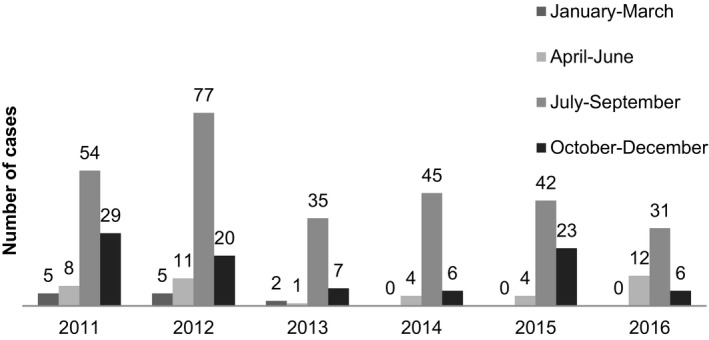
Season of hospitalized RSV‐associated LRTI in Bangkok, Thailand 2011‐2016

From 2011 to 2016, there were 427 hospitalized RSV‐associated LRTI patients in this study, and we stratified into 2 groups of community‐acquired RSV‐associated LRTI (n = 361 85%) and hospital‐acquired (nosocomial) RSV‐associated LRTI (n = 66, 15%). Median age of all patients was 10 (IQR 4‐23) months old. Majority of patients was 0‐6 months of age. Male was 45% of all patients. Less than quarter was pre‐term (21%). Half of the patients were having co‐morbidities. Most common co‐morbidities were chronic lung disease, congenital heart disease, and cirrhosis (19%, 16%, and 6%, respectively). Baseline demographic data are shown in Table 1. Peak of RSV‐associated LRTI was in July‐September of each year. Seasonal of RSV is shown in Figure 1.

**TABLE 1:** Baseline characteristics of patients under 5 y old hospitalized with RSV‐associated LRTI in Bangkok, Thailand, 2011‐2016

	Total N = 427	Not severe N = 253	Severe^a^ N = 174	*P*‐value
Age (mo), (P25th‐P75th)	10.8 (4.2‐23.0)	11.1 (4.2‐23.4)	8.8 (3.6‐19.8)	.09
Age group, N (%)				
0‐6 mo	135 (32)	74 (29)	61 (35)	.47
7‐12 mo	95 (22)	54 (21)	41 (24)	
1‐2 y	148 (35)	95 (38)	53 (31)	
3‐5 y	49 (12)	30 (12)	19 (11)	
Male, N (%)	194 (45)	110 (44)	84 (48)	.33
Preterm^b^, N (%)	88 (21)	40 (16)	48 (28)	.01
Co‐morbidities, N (%)	219 (51)	90 (35)	129 (71)	
Chronic lung disease	81 (19)	24 (9)	57 (33)	<.001
Congenital heart disease	67 (16)	22 (9)	45 (11)	<.001
Non‐cyanotic heart	37 (9)	10 (4)	27 (16)	
Cyanotic heart	30 (7)	12 (5)	18 (10)	
Cirrhosis/Biliary atresia	26 (6)	11 (4)	15 (9)	<.001
On chemotherapy	25 (6)	16 (6)	9 (5)	.56
Anemia	16 (4)	9 (4)	7 (4)	.95

Note

Data present median (IQR) and N (%), compare using chi‐square for compare category data and Wilcoxon rank‐sum test for continuous data.

^a^ Severe hospitalized RSV defined as patient who needs intubation or non‐invasive positive pressure ventilation (such as high flow nasal cannula, continuous positive airway pressure (CPAP), bi‐level positive airway pressure (BIPAP)), or in‐hospital death and prolong length of hospital stay in community‐acquired RSV infection (more than 7 d).

^b^ Gestational age < 37 wk.

#### 3.1.2 Clinical presentation of community‐acquired RSV‐associated LRTI

Most common clinical presentation for community‐acquired RSV‐associated LRTI were cough, fever, and dyspnea (91%, 87%, and 77%, respectively). From 361 patients, 46 patients (13%) had body temperature >38.7°C. Only 105 of 361 (29%) had desaturation (partial Oxygen saturation < 95%) at initial presentation, and 18 of 361 (5%) had congenital cyanotic heart disease. Nine patients had seizure prior to admission including 5 cases of febrile convulsion and 4 cases of epilepsy with fever provoke seizure. Of the 361 patients, 126 patients (35%) were documented of wheezing at first presentation (49 patients were severe, and 77 were not severe). However, fever, cough, dyspnea, poor intake, vomiting, and diarrhea were not different in severe or not severe hospitalized RSV‐associated LRTI (89% vs 85%, 99% vs 91%, 81% vs 83%, 71% vs 56%, 42% vs 28%, and 23% vs 16%, respectively). Fever at initial presentation was 37.7°C similarly in both groups. And peak of fever at 38.5°C during first few days was not different in severe and not severe RSV‐associated LRTI. Thrombocytosis was found in 95 of 361 patients (26%) including 46/361(13%) severe and 49/361(14%) not severe RSV‐associated LRTI, *P*‐value .1.

#### 3.1.3 Treatment of RSV‐associated LRTI

Oxygen therapy was prescribed in all patients, mainly low flow oxygen therapy (86%). Invasive ventilator support was used in 56 out of 427 patients, including 37 infants (16% of 230 infants) and 19 older children (10% of 197 patients age >1 year). Infants needed invasive ventilator support more than older children. Bronchodilator (salbutamol) was nebulized in 317/361 (88%) of all patients similar in both not severe and severe group (186/212 (87%) and 133/149 (89%), respectively). Of the 361 patients, 282 patients (78%) received salbutamol nebulizer for 2 days or more. Ceftriaxone and/or cefotaxime were the most common prescribed antibiotics from total 137/361 patients (38%). 94/149 (63%) of severe group were prescribed while only 43/212 (20%) in not severe group. Management of hospitalized community‐acquired RSV‐associated LRTI is shown in Table 2.

**TABLE 2:** Pattern of management of hospitalized community‐acquired RSV‐associated LRTI in Bangkok, Thailand, 2011‐2016

Community acquired	Total N = 361	Not severe N = 212	Severe N = 149	*P*‐value
Oxygen therapy, N (%)				
Low flow oxygen delivery^a^	300 (83)	211 (99)	89 (60)	<.001
High flow oxygen delivery	18 (5)	0 (0.0)	18 (12)	
Positive pressure ventilation^b^	6 (2)	0 (0.0)	6 (4)	
Invasive mechanical ventilation	37 (10)	0 (0.0)	37 (25)	
Bronchodilator nebulizer^c^, N (%)	317 (88)	184 (87)	133 (89)	.48
Intravenous antibiotic, N (%)	137 (38)	43 (20)	94 (63)	<.001
Ceftriaxone/cefotaxime	102 (28)	38 (18)	64 (43)	<.001
Piperacillin/tazobactam or ceftazidime	16 (4)	2 (1)	14 (9)	<.001
Ampicillin^d^	8 (2)	3 (1)	5 (3)	.22
Other^e^	11 (3)	0 (0)	11 (7)	<.001
Length of hospital stay (d), median (P25th‐P75th)	5 (3‐9)	4 (3‐5)	12 (8‐20)	<.001

Note

Data present median (ICR) and N (%), chi‐square for compare category data and Wilcoxon rank‐sum test to compare continuous data.

^a^ Low flow oxygen delivery including oxygen nebulizer, oxygen box, oxygen nasal cannula, oxygen nasal mask, and oxygen mask with bag.

^b^ Non‐invasive positive pressure ventilation including bi‐level positive airway pressure (BIPAP) and continuous positive airway pressure (CPAP).

^c^ Bronchodilator was salbutamol nebulizer.

^d^ Ampicillin including amoxicillin/clavulanic acid and ampicillin/sulbactam.

^e^ Other antibiotics were levofloxacin and meropenem.

#### 3.1.4 Outcome after hospital stay

Readmission rate within 1 month with the same diagnosis of RSV‐associated LRTI was 0.5%. There were 11 dead cases in this study (2.6%). All cases had co‐morbidities as presented in Table 3. From all dead cases, 6 cases (1.4% of 361 cases) were community‐acquired and 5 cases (7.6% of 66 cases) were hospital‐acquired RSV‐associated LRTI.

**TABLE 3:** Risk factor associated with severe community‐acquired RSV‐associated LRTI in Bangkok, Thailand, 2011‐2016

	Univariate		Multivariate	
	OR (95% CI)	*P*‐value	aOR (95% CI)	*P*‐value
Age < 1 y	1.18 (0.78‐1.81)	.44		
Female	1.27 (0.83‐1.93)	.27		
Preterm	1.86 (1.11‐3.1)	.02	2.03 (1.13‐3.67)	.02
Co‐morbidity				
Cardiovascular				
Cyanotic heart	3.28 (1.27‐8.47)	.01	0.24 (0.05‐1.18)	.08
Non‐cyanotic heart	6.06 (2.53‐14.51)	<.001	5.11 (1.97‐13.23)	.001
Pulmonary disease				
Chronic lung disease	9.02 (4.37‐18.61)	<.001	15.16 (4.26‐53.91)	<.001
GI disease				
Cirrhosis/BA	10.59 (2.33‐48.11)	<.001	15.01 (3.21‐70.32)	.001
Immunocompromised status				
Chemotherapy	3.37 (1.02‐11.15)	.05	4.70 (1.34‐16.56)	.02
Hematologic condition				
Iron deficiency anemia	0.70 (0.21‐2.39)	.57		
Thalassemia	1.41 (0.09‐22.72)	.81		

Abbreviations: 95% CI, 95% confidence interval; aOR, adjusted odds ratio; OR, odds ratio.

### 3.2 Factor associated with RSV‐associated LRTI

**FIGURE 2:**
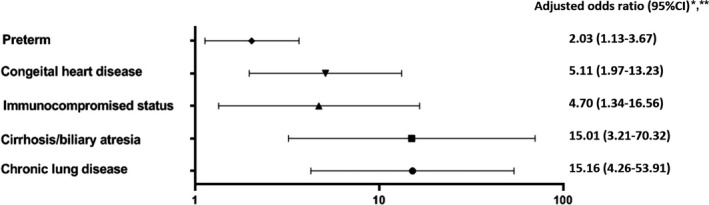
Factor associated with severe hospitalized RSV‐associated LRTI** in children under 5 y old in Bangkok, Thailand, 2011‐2016. *Data were analyzed by multivariate logistic regression model. **Severe hospitalized RSV defined as patient who needs intubation or non‐invasive positive pressure ventilation (such as high flow nasal cannula, continuous positive airway pressure [CPAP], bi‐level positive airway pressure [BIPAP]), or in‐hospital death and prolong length of hospital stay in community‐acquired RSV infection (more than 7 d)

Severe RSV‐associated LRTI was 174/427 (41%). From all 174 severe RSV‐associated LRTI patients, there were 149 community‐acquired severe RSV‐associated LRTI and 25 hospital‐acquired RSV‐associated LRTI. Eleven patients were in‐hospital death and would be discussed in next section. From community‐acquired RSV‐associated LRTI, 37/361 (10%) were needed mechanical ventilator support, 24/361 (7%) were needed non‐invasive positive pressure ventilation, and 82/174 patients (47%) were needed prolong hospital stay at least 7 days. Risk factors associated with severe RSV‐associated LRTI were perinatal history of low birthweight (aOR 1.85 CI 1.17‐2.9, *P*‐value .01), pre‐term (aOR 1.98 CI 1.15‐3.41, *P*‐value.014), cardiovascular disease (aOR 3.55 CI 1.89‐6.68, *P*‐value < .001), gastrointestinal disease (aOR 4.07 CI 1.8‐9.24, *P*‐value .001), pulmonary disease (aOR 4.11 CI 2.03‐8.33, *P*‐value < .001), and neurological disease (aOR 10.12 CI 2.71‐37.72, *P*‐value .001). Underlying diseases of cardiovascular mostly were congenital heart disease such as ventricular septal defect, single ventricle, etc Both cyanotic and non‐cyanotic heart disease were associated with severe (OR 3.28 CI 1.27‐8.47, *P*‐value .01 and OR 6.06 CI 2.53‐14.51, *P*‐value < .001). Majority of pulmonary disease was chronic lung disease. For gastrointestinal diseases, there were cirrhosis, biliary atresia, short bowel syndrome, chronic diarrhea, etc which cirrhosis/biliary atresia was associated with severe (OR 10.59 CI 2.33‐48.11, *P*‐value < .001). Neurological diseases were epilepsy, cerebral palsy. Immunocompromised status was mostly patient receiving immunosuppressive agent such as chemotherapy. Factor associated with severe RSV‐associated LRTI is shown in Figure 2. Immunocompromised status such as patient on chemotherapy and hematologic disorder was not different in both severe and not severe RSV‐associated LRTI.

### 3.3 Factor associated with death

In hospital death of 11/427 (3%) from 2011 to 2016, there were 6 community‐acquired and 5 hospital‐acquired. Age range of death was mostly during infant period. Mortality rate is higher in hospital‐acquired than community‐acquired RSV‐associated LRTI (5/66 (8%) and 6/361(2%), respectively). All of mortality cases were having co‐morbidities which were similarly in both community‐ or hospital‐acquired infection. Cardiovascular disease especially cardiac anatomical defect such as ventricular septal defect was as expected as majority as 5 out of 11 cases of dead cases follow by those with cirrhosis or biliary atresia (4 of 11 cases). More than half 6/11 of cases complicated by nosocomial bacterial infection with *A baumanii, Pseudomonas* spp., and *Steotrophomonas* spp. which leaded to lethal outcome. Characteristic of 11 children with mortality after RSV‐associated LRTI showed in Table 4.

**TABLE 4:** Characteristic of 11 children with mortality after RSV‐associated LRTI in Bangkok, Thailand, 2011‐2016

No.	Age	Co‐morbidity	Day of diagnosis	LOS (d)	Cause of dead
Community‐acquired RSV infection					
1	4 mo	CLD, Pre‐term 26 wk	—	27	*A baumanii* pneumonia
2	5 mo	Single ventricle, Total anomalous of pulmonary venous return	—	12	*Stenotrophomonas* pneumonia
3	7 mo	Cirrhosis	—	7	Acute respiratory failure
4	7 mo	Cirrhosis, pre‐term	—	12	Multi‐organ failure
5	8 mo	Miller‐Dieker syndrome, CLD, VSD, pre‐term 34 wk	—	93	*Pseudomonas* pneumonia
6	1 y	Down syndrome, VSD	—	52	*Pseudomonas* pneumonia with DIC
Hospital‐acquired RSV infection					
7	5 d	Preterm 32 wk	Day 37 of admission	46	Pulmonary hypertension and Pulmonary hemorrhage
8	2 mo	BA s/p Kasai's operation	Day 7 of admission	19	Septicemia with DIC
9	7 mo	BA s/p Kasai's operation	Day 26 of admission	36	*Stenotrophomonas* sepsis, Intraabdominal infection
10	10 mo	CLD, VSD, chronic heart failure,	Day 120 of admission	167	Severe ARDS with septic shock
11	1 y	Single ventricle, chronic heart failure	Day 13 of admission	10	Cardiogenic shock

Abbreviations: BA, biliary atresia; CLD, chronic lung disease; DIC, disseminated intravascular coagulation; LOS, length of hospital stay (d) after RSV diagnosis; VSD, ventricular septal defect.

### 3.4 Hospital‐acquired RSV‐associated LRTI

Nosocomial infection was 66/427 (15%). Hospital‐acquired RSV‐associated LRTI was related to more death (hospital‐acquired 5/66 (8%), community‐acquired 6/361(2%), p‐value 0.005), and severe outcomes as higher ICU admission (hospital‐acquired 16/66 (24%), community‐acquired 43/361(12%) *P*‐value .008), positive pressure and mechanical ventilator respiratory support (hospital‐acquired 18/66 (27%), community‐acquired 36/361(10%) *P*‐value < .001), and also longer length of hospital stay as median of 43 days (IQR 19‐84) compare with 6 days (IQR4‐10), *P* < .001 in community‐acquired RSV‐associated LRTI. Others than initial presentation and laboratory finding that were similar to community‐acquired RSV‐associated LRTI, antibiotics were being used in numbers more than community‐acquired RSV‐associated LRTI (hospital‐acquired 46/66 [71%], community‐acquired 160/361 [44%]).

## 4 DISCUSSIONS

Almost half RSV‐associated LRTI hospitalization were having severe symptoms. Infant is still at high risk of RSV‐associated LRTI as majority of RSV‐associated LRTI was children 0‐6 months old.^15^ Gastrointestinal disease such as cirrhosis or biliary atresia was another important risk factors for severe RSV‐associated LRTI including resulted as in‐hospital death and might be another group to consider for prophylaxis treatment. Other correlate findings for risk factors with previous study were co‐morbidities of prematurity, low birthweight, cardiopulmonary disease, gastrointestinal disorder, and neuromuscular impairment.^14^, ^16^ Many studies had pointed out other risk factor such as immunocompromised status and Down syndrome^17^, ^18^, ^19^ but were not statistically significant associated with severe RSV in our study. Our study found no significant different between male and female sex as in western countries which male as one of risk factor.^20^ Other risk factors were maternal smoking during pregnancy, multiparity, and birth during first half of RSV season which might lead to promote of smoking cessation or avoidance.^21^ Our nosocomial infection was 15% which is more than previous study in Cape town (22/226 (10%)), which might be explained from different study population and hospital setting and also lower age group of median age 2.8 months in previous study. However, mortality rate (3%) in this study was still lower than 29% from systemic review in 2016 which may result from different prevalence and infection control in each setting.^22^, ^23^

Peak of RSV‐associated LRTI during each year was July–September which is rainy season in Thailand also similar finding of rainy season in previous study in Thailand and in study in Pakistan but not in winter like in western region and different from east Asia such as China which season of RSV start in mid‐October.^4^, ^24^, ^25^, ^26^

Mortality rate was 3% in this study which is more than previous study in Thailand (0.7%) in 2008‐2011.^4^ Higher mortality rate would be explain from complexity in tertiary hospital which taken care of patient with more complex diseases. Particulary, higher mortality rate found in nosocomial RSV‐associated LRTI and patient with underlying diseases of cardiopulmonary or gastrointestinal disease such as cirrhosis and biliary atresia. Therefore, cirrhosis or biliary atresia should be considered as one of the risk factors of severe RSV‐associated LRTI that need prevention as well as patient with cardiopulmonary disease. In this era, many patients survive and live with their diseases. More patients with co‐morbidity may explain more in‐hospital dead either from their susceptible to infection or RSV that causes more severe symptoms. Hence, prevention of RSV is necessary. During vaccine development and study, palivizumab is one of medication use especially in infant with hemodynamic significant heart disease for RSV prophylaxis.^27^ Moreover, specific treatment for RSV‐associated LRTI need to be studied to improve outcome of treatment.^28^

Treatment for RSV‐associated LRTI was mainly depend on clinical presentation. Even though desaturation was found in only 28.5% of cases at initial presentation, oxygen therapy was being use in all cases for respiratory support and to decrease work of breathing similar to other study in children with medical complexity that required oxygen during illness.^29^ In our study, bronchodilator nebulizer and antibiotics were used which might not be useful and was not recommended by AAP guideline.^7^ Even without initial wheezing, wheezing might develop later during hospital stay and might improve since most of salbutamol nebulizer was being continuously used more than 1 day. Most of antibiotic use for RSV‐associated LRTI was ceftriaxone or cefotaxime which aimed to cover most of community‐acquired bacterial infection which could be *Staphylococcus aureus and Pseudomonas aeruginosa*.^30^ But specific bacterial co‐infection could not be proved due to limitation of specimen for bacterial identification.

### 4.1 Strength and limitation

Subjective information such as history of clinical presentation might be limitation of this study due to retrospective study but we also collect objective data such as physical examination and laboratory findings that were recorded as first presentation. Clinical diagnosis of RSV‐associated LRTI was based on attending physician's medical record. Analysis of antibiotic use was limited due to secondary bacterial infection may be suspected in some cases but evidence of bacterial infection was not available in most cases.

## 5 CONCLUSION

RSV‐associated LRTI is high burden disease. Infants or children with co‐morbidities especially cardiopulmonary and biliary atresia have higher risk of severe RSV‐associated LRTI. No specific treatment is available so treatment is mainly supportive with oxygen therapy, bronchodilator, or other treatment based on clinical presentation and treatment response/outcome.

## CONFLICT OF INTEREST

Potential conflicts of interest. All authors: No conflicts.

## AUTHOR CONTRIBUTION

**Puneyavee Aikphaibul:** Conceptualization (equal); Formal analysis (supporting); Validation (equal); Writing‐original draft (lead). **Thanyawee Puthanakit:** Conceptualization (equal); Supervision (lead); Writing‐review & editing (lead). **Tuangtip Theerawit:** Resources (equal); Software (lead). **Jiratchaya Sophonphan:** Formal analysis (lead); Resources (equal); Writing‐original draft (supporting). **Noppadol Wacharachaisurapol:** Resources (equal); Writing‐original draft (supporting). **Nattapong Jjitrungruengnij:** Resources (equal); Writing‐original draft (supporting).
